# Supplementary material for: Telemedicine in Care of Sarcoma Patients beyond the COVID-19 Pandemic: Challenges and Opportunities
Source: Cancers (Basel). 2023 Jul 21;15(14):3700. doi: 10.3390/cancers15143700 (PMC10378403; doi:10.3390/cancers15143700)
Supplement: Supplementary file 1 [file cancers-15-03700-s001.zip › cancers-2477149-supplementary.pdf]

Supplementary material

Table S1: Overview of the eligible studies' characteristics

FIRST AUTHOR (STUDY)	DATE	STUDY TYPE	CANCER TYPE	SAMPLE	TYPE OF TELEMEDICAL INTERVENTION	PHASE OF CANCER CARE	CATEGORIZATION	DOI
McCabe et al.	2021	Comparative questionnaire-based survey, observational study	Sarcoma	74 patients, 26 providers	Telephone or video consultation	Under treatment, FU	TR, TO	10.1200/GO.20.00599
Younger et al.	2020	Cross-sectional questionnaire-based survey	Sarcoma	350 patients	N/A	Under treatment	Mental health, Satisfaction	10.3390/cancers12082288
Lawrenz et al.	2021	Cohort-telephone survey	Sarcoma	64 patients	Telephone	FU	Satisfaction, TO	10.3928/01477447-20210819-09
Hassan et al.	2021	Case report-observational	Leiomyosarcoma	1 patient	N/A	Diagnosis	Diagnosis, TO	10.14740/wjon1393
Olshinka et al.	2020	Retrospective observational study	Sarcoma	155 FUs, 96 new referrals	Email-fax-telephone	New referrals, FU	Surgery, TO	10.3747/co.27.6907
Yeshayah et al.	2021	Case series	Ewing Sarcoma	4 patients	Telephone consultation before ER	New referrals	Diagnosis	10.5041/RMMJ.10431
Rajasekaran et al.	2021	Questionnaire-based survey	Sarcoma	36/39 responses	Videoconferencing platforms	MDT	Satisfaction, TO	10.1186/s12891-020-03925-8
Pushpam et al.	2020	Letter to the editor	Various types including sarcoma	N/A	Telephone, email	Under treatment	TO	10.1002/pbc.28428
Natesan et al.	2021	Questionnaire-based survey	Various types including sarcoma	18 radiation oncology providers	Telephone or video	Under treatment	Satisfaction, teleradiology (TO)	10.1016/j.ijrobp.2021.07.1063
Cavaliere et al. (SICO)	2021	Review (guidelines)	Various types including STS	N/A	Videoconferencing platforms	Screening, FU, counseling	Surgery guidelines, TO	10.1007/s13304-020-00921-4
Spiess et al.	2020	Commentary	N/A	N/A	N/A	N/A	Benefits, challenges and opportunities	10.1002/cncr.32919
M van Erkel et al.	2022	Qualitative study using semi-structured interviews and reflexive	Various types including sarcoma	82 patients and 58 providers	Telephone consultation before ER	FU	Satisfaction, TO	10.1136/bmjopen-2021-058361

		thematic analysis						
Moreira et al.	2022	Editorial	Various types in children including sarcoma	N/A	N/A	N/A	TH in oncology and covid19, Diagnosis	10.1002/cncr.33946
Mohapatra et al.	2022	Retrospective observational study	Leukemia	249 children and adolescent patients	N/A	N/A	TH in oncology and covid19, Diagnosis	10.1080/08880018.2022.2025963
Turner et al.	2022	Qualitative study	N/A	40 providers	Real-time videoconferencing, virtual check-ins, telephone	N/A	Satisfaction	10.2196/29635
Folsom et al.	2021	Case series	Various types including sarcoma	2 patients	videocall (zoom)	Under treatment, FU	Satisfaction, mental health	10.1177/153473542110 53647
Nahshon et al.	2021	Systematic review	Various types	88 cancer patients	N/A	Under treatment	Diagnosis, TH in oncology and covid19	10.1080/1120009X.2021.1899442
De Vito et al.	2020	Editorial	N/A	N/A	N/A	N/A	Diagnosis, TH in oncology and covid19	10.26355/eurrev_202012_24219
Lidington et al.	2020	Retrospective, observational study	Sarcoma	350 patients	N/A	Under treatment	TR, TO	10.1002/cncr.33215
Buonaguro et al.	2020	Retrospective, observational study	Various types including sarcoma	N/A	Telephone, e-mail, a home health care platform, Intensive health care platform	Under treatment, FU	TH in oncology and covid 19, TO	10.1186/s13027-020-00330-7
Lenihan et al. (ICOS)	2020	Review	Various types	N/A	N/A	N/A	TO, guidelines	10.3322/caac.21635
Martinez et al. American Society for Radiation Oncology	2020	Questionnaire-based survey	Various types including sarcoma	115 responses	N/A	Under treatment, FU	Teleradiology (TO)	10.1016/j.ijrobp.2020.06.058
De Joode et al.	2020	Survey-based analysis	Various types including sarcoma	5302 patients	Telephone, video	Under treatment, FU	Satisfaction, mental health	10.1016/j.ejca.2020.06.019

Bogani et al.	2020	Review	Gynaicological cancer	N/A	various softwares	FU, second opinion	Surgery	10.3802/jgo.2020.31.e72
Košir et al.	2020	Cross-sectional study	Various types (adolescent and young adult malignancies)	177 individuals	N/A	Under treatment	TO, Mental health	10.1002/cncr.33098
Martin-Broto et al. (SELNET)	2020	Review (guidelines)	Sarcoma	N/A	N/A	Diagnosis, under treatment	Surgery guidelines, TO	10.1634/theoncologist.2020-0516
Burki et al.	2020	Commentary	Sarcoma	N/A	N/A	N/A	Surgery guidelines, TO	10.1016/S1470-2045(20)30217-5
Van de Haar et al.	2020	Review	N/A	N/A	N/A	N/A	TO	10.1038/s41591-020-0874-8
Callegaro et al.	2021	Review	GIST or STS	N/A	N/A	N/A	Surgery, TO	10.1002/jso.26246
Janssens et al. (SIOPE radiation oncology group)	2020	Review (guidelines)	Various pediatric tumors including sarcoma	N/A	N/A	Under treatment	Teleradiology (TO), guidelines	10.1016/j.radonc.2020.04.035
Ben-Arye et al. (SIO)	2021	Review, questionnaire, (guidelines)	N/A	N/A	Online consultation	Under treatment	TO, guidelines	10.1007/s00520-021-06205-w
West et al.	2022	Review	N/A	N/A	Telephone and/or video platforms	N/A	TO	10.1007/s11912-022-01332-x
Smith et al.	2021	Umbrella review	N/A	N/A	N/A	N/A	Surgery	10.1111/ans.17217
Llyod et al.	2022	Review	Hematologic malignancies	N/A	Telephone and/or video platforms	N/A	Benefits, challenges and opportunities, TO	10.1007/s11899-021-00642-4
Murphy et al.	2022	Systematic review	Various types	N/A	N/A	Under treatment, FU, rehabilitation, palliative care	Benefits, challenges and opportunities, TO, TR	10.3390/s22093598

Singh et al.	2021	Systematic review	Various types including sarcoma	N/A	Telephone and/or video platforms	Under treatment, FU	Satisfaction, mental health	10.3390/curroncol28050301
Siavashpour et al.	2021	Systematic review	Various types including sarcoma	N/A	N/A	Under treatment	Teleradiology (TO), guidelines	10.1016/j.critrevonc.2021.103402
McGrowder et al.	2021	Review	Breast cancer	N/A	Audio and videoconferencing	Screening, diagnosis, under treatment, rehabilitation	Satisfaction, TO	10.3390/healthcare9101401
Montenegro et al.	2020	Review	N/A	N/A	Tele-tumor board	Under treatment, palliative care	TH in oncology and covid19	10.1016/j.critrevonc.2020.103129
Joshua P Kronenfeld and Frank J Penedo	2021	Review	Various types	N/A	N/A	N/A	Benefits, challenges and opportunities	10.1093/tbm/ibaa105
Shaikh et al.	2022	Systematic review	N/A	N/A	N/A	N/A	Surgery, TO	10.1016/j.jsurg.2022.04.015
Naik et al.	2022	Review	N/A	N/A	Virtual consultations	N/A	TH and covid19	10.3389/fdgth.2022.919985
Baldwin et al.	2022	Review	N/A	N/A	N/A	N/A	Surgery, TO	10.1188/22.CJON.374-382
Lu et al.	2022	Systematic review	Various types	N/A	Mobile health applications	Under treatment, FU	TO	10.1016/j.adro.2020.09.016
Panet et al.	2022	Review and 4 cases	various types	4 cases	N/A	Diagnosis, under treatment	Satisfaction, TO	10.1002/cnr2.1531
Lammers et al.	2017	Retrospective observational study	N/A	N/A	N/A	N/A	History of TH	10.1111/1475-6773.12550
Lam et al.	2020	Cross-sectional study	N/A	N = 4525	telephone-video visit	N/A	Challenges and opportunities	10.1001/jamainternmed.2020.2671
Shah et al.	2020	Review	N/A	N/A	Telephone, video consultation	Diagnosis, under treatment	TO	10.3322/caac.21627
Ramaswamy et al.	2020	Retrospective, observational cohort study	N/A	N/A	Videovisits	N/A	Benefits	10.2196/20786

Levit et al.	2020	Review	N/A	N/A	N/A	N/A	Benefits	10.1200/OP.20.00174
Blake et al.	2017	Retrospective observational study	N/A	18 cancer registries	N/A	N/A	Benefits	10.1158/1055-9965.EPI-17-0092
Global Health Research Group on Children’s Non-Communicable Diseases Collaborative	2022	Retrospective observational cohort study	Various pediatric tumors including sarcoma	1660 patients (91 hospitals and cancer centres in 39 countries)	N/A	New referrals, under treatment	TO	10.1136/bmjopen-2021-054690
Onesti et al.	2022	Retrospective study	STS and bone sarcomas or aggressive benign musculoskeletal diseases	372 patients	N/A	Diagnosis	TR, TO	10.3389/fonc.2022.1000056
Sarfraz et al.	2022	Retrospective study	Ewing sarcoma	20 patients	N/A	Under treatment	TO	10.1002/pbc.29595
Smrke et al.	2020	Observational, questionnaire-based survey	Sarcoma	316 patients (379 appointments), 18 providers and 108 patients completed the survey	Telephone	New referrals, under treatment, FU	Satisfaction, TO	10.1200/GO.20.00220
Jiang et al.	2021	Review	Various types	N/A	telephone, or video consultation	Diagnosis, under treatment, FU	TO	10.1200/OP.20.00520
Jiménez-Rodríguez et al.	2020	Descriptive, qualitative, observational study	N/A	53 professionals	Telephone or videoconference, telemonitoring/screening	N/A	Satisfaction, TO	10.3390/ijerph17145112
Chowdhury et al.	2020	Review	Various types	N/A	N/A	Rehabilitation,palliative care	TO	10.1016/j.jpainsymman.2020.07.030
Pareek et al.	2020	Review	Various types	N/A	Audiovisual information technology for	Triage, counseling, FU	TO	10.1200/GO.20.00295

					interaction or monitoring-applications for chatting and video calls, chatbots with artificial intelligence, and various software (Skype and Cisco Webex)			
Ignatowicz et al.	2019	Review	Various types	N/A	Video-consultations	Under treatment, rehabilitation	TR, TO	10.1177/2055207619845831
Shaverdian et al.	2021	Questionnaire-based survey	Various types	TH: 351/1077 radiation oncology patients	TH consultation	Under treatment	Satisfaction, teleradiology (TO)	10.6004/jnccn.2020.7687
Isautier et al.	2020	Cross-sectional survey	N/A	1369 patients	Telephone, videoconference	N/A	Satisfaction	10.2196/24531
Onesti, Tagliamento et al.	2021	Questionnaire-based survey	N/A	109 medical oncologists	N/A	N/A	Satisfaction and TO	10.1200/GO.20.00589
Patt et al.	2021	Observational study	N/A	640 practitioners at 221 sites of service	Multidisciplinary TH strategy	Under treatment, FU	TH in oncology and covid19 , TO	10.1200/OP.20.00815
Gutkin et al.	2020	Questionnaire-based survey	Breast, gastrointestinal	56 patients	Video visits	Under treatment, FU	Satisfaction, Teleradiology (TO), challenges and opportunities	10.1016/j.ijrobp.2020.06.047
Darcourt et al.	2021	Questionnaire-based survey	Various types	1477/1762 patients participated	Video visits	Under treatment	Satisfaction, TO	10.1200/OP.20.00572
Lorgelly et al.	2020	Commentary	Various types	N/A	health technology assessment	N/A	TH and covid19	10.1007/s40258-020-00590-9
Bland et al.	2020	Point of view, uncorrected manuscript	N/A	N/A	N/A	N/A	TR, exercise, compliance	10.1093/ptj/pzaa141
Larson et al.	2020	Systematic review and metanalysis	Cancer survivors	N/A	N/A	FU (survivors)	Satisfaction	10.1177/1460458219863604

Aapro et al.	2020	Review	Various types	N/A	38 digital solutions	Palliative care	Rehabilitation, physical exercise	10.1007/s00520-020-05539-1
Cormie et al.	2017	Systematic review	Various types	100 studies	N/A	N/A	Rehabilitation, physical exercise	10.1093/epirev/mxx007
Campbell et al.	2019	Review (practice guidelines)	Cancer survivors	N/A	N/A	FU (survivors)	Rehabilitation, physical exercise	10.1249/MSS.0000000000002116
Garcia et al.	2014	Review	Cancer survivors	N/A	N/A	FU (survivors)	Rehabilitation, physical exercise	10.1177/0884533614551969
Tarasenko et al.	2018	Observational study	Cancer survivors	13997 observations	Muscle-strengthening activities and/or aerobic activities	FU (survivors)	Rehabilitation, physical exercise	10.1007/s10552-018-1017-0
Eng L et al.	2018	One-time questionnaire-based retrospective study	Cancer survivors	1003 patients	Physical activity	Under treatment, FU (survivors)	Rehabilitation, physical exercise	10.1007/s00520-018-4239-5
Schade et al.	2020	Opinion article	Various types	N/A	N/A	N/A	Mental health, TO	10.2217/fon-2020-0552
Hulbert-Williams et al.	2021	Prospective cross-sectional study	Various types (predominantly: breast cancer)	144 patients	N/A	FU, palliative care	Mental health, satisfaction	10.1111/ecc.13442
Malliaras et al.	2020	Pilot randomized controlled trial	N/A	36 of 38 (95%) eligible participants	Rotator Cuff-Related Shoulder Pain	FU	TR	10.2196/24311
McCue et al.	2010	Review	N/A	N/A	N/A	FU	Quality of life, benefits, TR, satisfaction	10.1016/j.pmr.2009.07.005
Hasson et al.	2021	Satisfaction questionnaire-based survey	Gastrointestinal	172/236 patients (74%) agreed to participate	TH meeting	Under treatment, follow -up	TO, satisfacrtion	10.1002/onco.13676
Loree et al.	2021	International Internet-based cross-sectional survey	Colorectal, breast, prostate and lung	381 patients	Virtual oncology appointments	Under treatment, FU	TO, satisfaction	10.3390/curroncol28010065
Berlin et al.	2021	Observational study	Various types	3507 patients and 284 practitioners, 22 085 Virtual Care visits	Virtual care program	Under treatment	TO, satisfaction	10.1001/jamaoncol.2020.6982

Khairat et al.	2020	Cohort study	N/A	92 confirmed COVID-19 cases and 733 total virtual visits.	N/A	N/A	TO, TH and covid	10.2196/18811
Vecchione et al.	2020	Review	Colorectal cancer	N/A	N/A	Diagnosis, under treatment	TO	10.1136/esmoopen-2020-000826
Orazem et al.	2020	Quantitative questionnaire-based survey	Various types	468 patients and 101 physicians	Video call	Under treatment and FU	TO, satisfaction	10.1016/j.ijrobp.2020.06.052
Onesti et al.	2020	Observational study	Various types	30 oncological centres	N/A	Under treatment	TO	10.1136/esmoopen-2020-000853
Sabesan et al.	2012	Questionnaire-based telephone survey	various types	55 patients	Videoconference	Under treatment, FU	TO, satisfaction, benefits	10.1111/j.1445-5994.2011.02537.x
Sabesan et al.	2014	Commentary	N/A	N/A	N/A	N/A	TO, benefits	10.1111/ecc.12251
Paterson et al.	2020	Review	N/A	N/A	N/A	N/A	TO, TH and covid19	10.1016/j.soncn.2020.151090
Morrison et al.	2020	Review	N/A	3698 participants	TH platforms such as Zoom, Skype, Microsoft Teams, or FaceTime	N/A	TO, TH and covid19	10.1016/j.soncn.2020.151092
Narayanan et al.	2021	Cohort study	Various types including sarcoma	509 telephone consultations to new patients vs 842 new patients in-person	zoom platform	Counseling	TO, TH and covid19	10.1177/15347354219 9101
Steindal et al.	2020	Scoping systematic review	N/A	N/A	N/A	Palliative care	TO, TH and covid19 , TR	10.2196/16218
Naidich et al.	2020	Retrospective study	N/A	N/A	N/A	Diagnosis	TO, diagnosis, TH and covid19	10.1016/j.jacr.2020.05.004
Norbash et al.	2020	Comparative study	N/A	N/A	N/A	Diagnosis	TO, diagnosis, TH and covid19	10.1016/j.jacr.2020.07.001
Greenwald et al.	2017	Systematic review	Various types	78 studies	Mobile screening units	Diagnosis	TO, diagnosis, TH and covid19	10.1158/1055-

								9965.EPI-17-0454
Al-Shamsi et al.	2020	Review	N/A	N/A	N/A	Under treatment	TO, surgery	10.1634/theoncologist.2020-0213
Hazin et al.	2010	Review	N/A	N/A	N/A	Under treatment	TO	10.1016/S1470-2045(09)70288-8
Sirintrapun et al.	2018	Review	N/A	N/A	N/A	Under treatment, FU	TO	10.1200/EDBK_200141
Sabesan et al.	2014	Review	Various types	N/A	N/A	N/A	TO, satisfaction, benefits	10.1111/ajco.12225
Tiwari et al.	2022	Review	Bone sarcoma	N/A	N/A	Under treatment	TO, surgery	10.7759/cureus.25245
Chazan et al.	2020	Multicenter observational study	N/A	Medical oncologists (n=372; 74%), radiation oncologists (n=91; 18%) and surgical oncologists (n=38; 8%).	N/A	Under treatment	TO	10.1136/esmoopen-2020-001090
Prajapati et al.	2021	Ambi-directional cohort	N/A	473 patients	Teleconsultation	Under treatment	TO, surgery	10.1016/j.jcot.2021.101651
Rossi et al.	2020	Observational study	N/A	N/A	N/A	Under treatment	TO, surgery	10.3390/jcm9061868
Zaniboni et al.	2020	Review	N/A	N/A	N/A	Under treatment	TO, surgery	10.3390/cancers12092452
Marrari et al.	2023	Opinion article	Sarcoma	N/A	N/A	Under treatment	TO, surgery	10.1097/CEJ.0000000000000692
Casali et al.	2018	Review (ESMO Practice Guidelines for diagnosis, treatment and FU)	bone sarcomas	N/A	N/A	Under treatment, FU	TO, surgery, guidelines	10.1093/annonc/mdy310
Strauss et al.	2021	Review (ESMO Practice Guidelines for diagnosis, treatment and FU)	bone sarcomas	N/A	N/A	Under treatment, FU	TO, surgery, guidelines	10.1016/j.annonc.2021.08.1995

Casali et al.	2022	Review (ESMO Practice Guidelines for diagnosis, treatment and FU)	GIST	N/A	N/A	Under treatment , FU	TO, guidelines	10.1016/j.annonc.2021.09.005
Curigliano et al.	2020	Review (ESMO practice guidelines)	Various types	62 global experts and one patient advocate	N/A	Under treatment , FU	Surgery, TO, guidelines	10.1016/j.annonc.2020.07.010
Catanese et al.	2020	Review (ESMO Management and treatment guidelines)	Pancreatic cancer	N/A	N/A	Under treatment , FU	Surgery, TO, guidelines	10.1136/esmoopen-2020-000804
Imlach et al.	2020	Online survey	N/A	38 interviewees	TH consultations	Under treatment	TO, satisfaction	10.1186/s12875-020-01336-1
Drerup et al.	2021	Comparative observational survey	N/A	65 patients: TH visits, 36: in-office visits	TH visits and video	N/A	TH and covid19, benefits, satisfaction	10.1089/tmj.2021.0002
Barney et al.	2020	Observational study	N/A	N/A	N/A	N/A	TH and covid19	10.1016/j.jadohealth.2020.05.006
Gao et al.	2020	Review and metanalysis	N/A	N/A	N/A	N/A	TH and covid19	10.21037/atm-20-3315
Chauhan et al.	2020	Joint Position Statement	N/A	N/A	N/A	N/A	TH and covid19 , benefits	10.4103/JETS.JETS_32_20
Cox et al.	2017	Systematic review	Cancer survivors	N/A	N/A	FU (survivors)	TR, TO	10.2196/jmir.6575
Marzorati et al.	2018	Systematic review	Various types	N/A	Web-based platforms and telephone calls	Palliative care	Satisfaction	10.2196/jmir.9812
Mahoney et al.	2020	Case report	N/A	3 nurses	N/A	Palliative care	Satisfaction, benefits	10.1097/WON.0000000000000694
Melillo et al.	2022	Narrative review	Cancer survivors	N/A	N/A	FU (survivors)	TR, benefits	10.3390/cancers14133163
Reynolds et al.	2022	Pilot study (randomized controlled trial)	Breast cancer	38 female patients	Immersive virtual reality	Palliative care	TR, satisfaction, benefits	10.1186/s12885-021-09081-z
Neil-Sztramko et al.	2019	Systematic review	Breast cancer	N/A	Physical activity	FU (survivors)	TR, physical exercise	10.1136/bjsports-2017-098389
Lopez et al.	2021	Multimethod study	Various types	1968 patients visits, 12 survivors and 12 oncology health care	Virtual care	FU	TR, benefits	10.1016/j.apmr.2021.02.002

				providers. 168 patients were assessed virtually.				
COVIDSurg Collaborative	2020	Global expert response study	N/A	N/A	N/A	Under treatment	Surgery	10.1002/bjs.11746
COVIDSurg Collaborative	2021	International, prospective, cohort study	15 tumor types	20006 adults	N/A	Under treatment	Surgery	10.1016/S1470-2045(21)00493-9
Chirico et al.	2016	Systematic review	N/A	N/A	Virtual care	N/A	TR	10.1002/jcp.25117
Shen et al.	2021	Review	N/A	N/A	Digital technology-based TH platform	N/A	Benefits	10.3389/fmed.2021.646506
Lieneck et al.	2020	Review	N/A	N/A	N/A	N/A	Benefits, challenges and opportunities, TH and covid	10.3390/healthcare8040517
Khera et al.	2017	Review	Hematologic malignancies	N/A	N/A	Under treatment	Benefits, challenges and opportunities	10.1182/bloodadvances.2017008789
Williams et al.	2018	Narrative review	N/A	N/A	N/A	N/A	Benefits, challenges and opportunities, TR	10.21037/mhealth.2018.04.03
Yildiz et al.	2020	Descriptive cross-sectional study	Various types (majority: breast cancer)	421 patients	Voice call	FU (survivors)	TO, benefits	10.2217/fon-2020-0714
Wagner et al.	2022	CCC19-Registry Based Retrospective Cohort Study	Sarcoma	281 patients	N/A	N/A	TO, sarcoma and covid	10.3390/cancers14174334
Teeyapun et al.	2022	Prospective, multicenter observational study	Solid malignancies	385 patients	N/A	N/A	TO, sarcoma and covid19, vaccines	10.1016/j.eclinm.2022.101608
Guimaraes de Sousa et al.	2022	Case report	Salivary gland myoepithelial	1 patient	N/A	N/A	TO, sarcoma and covid19, vaccines	10.1136/jitc-2021-004371

			carcinoma					
Quintero et al.	2022	Case report	Granulomatous mass mimicking a sarcoma	1 patient	N/A	N/A	Sarcoma and covid19, vaccines	10.1016/j.radcr.2022.05.035
Hong Ryu et al.	2023	Retrospective trial	Sarcoma	5927 (223 patients eligible)	N/A	New referrals	TO	10.1002/jso.27237
Prabowo Putro et al.	2023	Meta-analysis	Bone sarcomas and STS	N/A	N/A	New referrals	Surgery	10.1016/j.jor.2023.02.013
Kotrych et al.	2022	Retrospective study	Bone sarcomas	87 patients	N/A	New referrals	Diagnosis	10.3390/cancers14246037

Table S1 Overview of the 135 eligible studies' characteristics. CCC19-registry: COVID-19 and Cancer Consortium Registry, ER: emergency room, FU: Follow-up, GIST: Gastrointestinal stromal tumor ICOS: International Cardio-Oncology Society, N/A: Not applicable, SELNET: Sarcoma European and Latin American Network, SICO: Italian Society of Oncological Surgery, SIO: Society for Integrative Oncology, SIOPE: European Society of Paediatric Oncology, STS: Soft-tissue sarcomas, TH: Telehealth, TO: Tele-oncology, TR: Telerehabilitation
